# Results of the Cholera in 1854

**Published:** 1855-03

**Authors:** 


*Results of the Cholera in* 1854.—The cholera in 1848–49 (15 months) was fatal to 14,593 persons; in the last epidemic, extending from August, 1853, to November, 1854 (16 months) 11,495 persons fell victims. Allowing for increase of population, the deaths to every 10,000 living give an average of 64 in the former, and 46 in the latter. By cholera and diarrhœa together the deaths were in

| | | |
|---|---|---|
| 1848—49 | 18,431 | 81 in 10,000. |
| 1853—54 | 15,762 | 63 in " |

*Disparity between the Districts.*—Two (the north and central) suffer a mortality of only 16 in 10,000 living, while the south has 93 of every 10,000 cut off by the disease. In the report on cholera in 1849, by the registrar-general, it is said that the elevation of the soil in London has a more constant relation with the mortality from cholera than any other known element—the mortality being in the inverse ratio to the elevation; and so exactly has this been verified in the present epidemic that a scale of premium might have been drawn out in 1849 to rule in 1854 to the following effect: For a person of average condition, dwelling under 20 feet of elevation, the premium to insure £1,000 would be £12; while from those living at from 100 to 350 feet elevation the life office would be secure with a £2 premium.

The following facts, worked out by the registrar general, show distinctly the inverse relation that the mortality of cholera bears to the elevation of the ground:

182,560 of the people of London, in 1851, lived upon sub-districts covering 2,849 acres of the marsh ground, ranging from three feet below to 1 foot above the high-water mark; 2,693 died there of cholera in 1849, and 2,227 in 1854, or 4920 in the two epidemics.

263,914 of the population, in sub-districts on 13,146 acres of ground of 80 feet of elevation and upward, lost 398 persons by cholera in 1849, and 272 in 1854, or 670 in two epidemics.

12,824 persons died of cholera in the two years 1849 and 1854 on the 18,429 acres of low ground under 1[illegible] feet of elevation, out of a population of 595,119; while in the same years, out of the more numerous population, 682,706 persons, living on 21,909 acres of the higher ground of 60 feet and upward, only 2,949 persons died of cholera, including all the deaths in the district of St. James.

On the lowest ground, taking the mean of the two epidemics, 13 in 1,000 of the population—on the highest ground 1 in 1,000 of the population, were destroyed by cholera.

At the intermediate stages of elevation was the danger of dying by cholera intermediate? To solve this important question, as regarded the epidemic of 1849, London was first sub-divided into terraces differing twenty feet in elevation; and, if the same course is pursued now, it is found that in the two epidemic years 15,562 persons died of cholera on the first terrace, under twenty feet of elevation; 3,757 on the second terrace of ground, twenty feet and under forty feet high; 2,301 on the third terrace, forty and under sixty feet; 2,279 on the fourth terrace, sixty to eighty feet high; 392 on the fifth terrace, eighty to 100 feet; 278 on the higher terraces, of 100 feet up to 350 feet. The population was 850,000 on the lowest terrace; and about equal, or 400,000, on the second, the third, and the fourth terraces; while it was 142,000 on the fifth, and 121,000 on the higher terrace or terraces.—*Medical Times and Gazette.*

